# Children’s Access to Non-School Destinations by Active or Independent Travel: A Scoping Review

**DOI:** 10.3390/ijerph191912345

**Published:** 2022-09-28

**Authors:** Elise Desjardins, Zahra Tavakoli, Antonio Páez, Edward Owen Douglas Waygood

**Affiliations:** 1 School of Earth, Environment & Society, McMaster University, Hamilton, ON L8S 4K1, Canada; 2 Department of Civil, Geological and Mining Engineering, Polytechnique Montréal, Montréal, QC H3T 1J4, Canada

**Keywords:** active travel, activity spaces, children, destination, independent travel, meaningful places, territorial range

## Abstract

Background: Children’s access to non-school destinations is important for their well-being, but this has been overlooked in transport planning. Research on children’s access to non-school destinations is growing, and there is a need for a comprehensive overview, examining both quantitative and qualitative studies, of the existing evidence on places that children access by active or independent travel. Objectives: Identify and summarize quantitative and qualitative research on the topic of active or independent travel to non-school destinations for elementary aged children (6 to 13 years old). Methods: Papers published in English between 1980 and July 2021 were sourced from: (i) Web of Science Core Collection; (ii) PubMed; and (iii) APA PsycInfo. Three relevant journals related to children and transport were hand searched: (i) Children’s Geographies; (ii) Journal of Transport & Health; and (iii) Journal of Transport Geography. The search was limited to peer-reviewed articles published in English between 1980 and July 2021. Covidence, an online software platform for systematic reviews, was used to organize articles during the title and abstract screening stage. PRISMA-Scr is applied for reporting. Results: 27 papers were retained from an initial 1293 identified peer-reviewed articles. The results reveal that children in different geographies travel unsupervised or by active modes to places that support different domains of their well-being such as a friend or relative’s home, local parks or green spaces, recreational facilities, and different retail locations (e.g., restaurants). There is evidence that children’s ability to reach certain places is constrained, likely due to safety concerns or environmental barriers. Conclusions: Research on children’s diverse destinations is relatively limited as compared to trips to school. Various methodologies have been applied and can be combined to completement each other such as objective GPS tracking and subjective surveys on places children would go if they were available. Future research should clearly report and discuss the non-school destinations that children access to better inform transport planning and policy for all aspects of children’s lives.

## 1. Introduction

Active or independent travel among children has been increasingly promoted in many urban environments for several decades to reduce growing rates of childhood obesity and increase daily opportunities for physical activity. A decline in children’s active or independent travel has been observed, especially between generations [1,2]. Walking rates to school among children have declined sharply in North America [3], and some studies speak of “inactivity” or “immobility” when discussing children’s travel [2]. For these reasons, the desire to address this public health issue is warranted. In several ways, children’s independent mobility (CIM), or making trips without adult supervision, is beneficial. Having the opportunity to engage in independent travel in their neighborhood leads to children socializing more frequently with their peers and adults, and they develop social skills as a result [4,5]. In the absence of adult supervision, children can learn from various interactions with their surroundings and experiences with other people. Active or independent travel help to promote spatial awareness [6], learning about risk and how to manage it [7], and engaging with the natural and physical environment [8] which all improve children’s cognitive and psychological development.

Children’s long-term well-being is associated with such behaviors and experiences since they contribute to mental/psychological, physical, and social development [9]. There are five domains of well-being that relate to transport for children [10]: physical (i.e., physical activity, exercise), psychological (i.e., mental or emotional health), cognitive (i.e., learning, exploring), social (i.e., social interactions, social capital, social skills, connections to community), and economic (i.e., support for children to access destinations). For optimal well-being, children should be able to travel to a variety of non-school destinations that contribute to different domains of their well-being (e.g., green spaces, recreational facilities, libraries, etc.) ideally through active or independent means to derive additional intrinsic benefits from transport itself. Waygood et al.’s [10] integrative review demonstrates that independent travel provides access to destinations important for well-being and creates opportunities for exploration and socialization which support children’s cognitive and social needs.

Research indicates that different factors at the socioecological level limit children’s active and independent travel in urban environments around the world. Increasing traffic volumes and parental concerns about traffic safety have led to children being increasingly escorted, mostly by car [2]. The design of streets and the presence of sidewalks and crosswalks are crucial [11,12]. A range of household characteristics, including socioeconomic status and beliefs or practices about mobility, influence how children’s travel. For example, a parental preference for living in areas with less intensive development can limit a child’s ability to travel to a diversity of places because the distance threshold is too far [13]. Personal security must, however, be considered in a safety context as well. As an example, with the concept of ‘eyes on the street’, the quality and size of the residential blocks and the number of street-level retailers in the neighborhood may contribute to children’s safety during their independent travel [14,15]. Moreover, a lack of safety and security could cause children to make more dependent trips and walk less [11,16,17]. According to previous research, children walk less in neighborhoods with ‘stranger danger’ concerns [11]. Therefore, children who have strong community and social cohesion can meet others and are encouraged to be independent when traveling to their daily destinations [11,16]. Culture also plays a role in explaining differences in children’s travel and independence [18].

To date, children’s active travel or independent mobility to school has primarily been the policy focus. This topic has also been studied quite extensively [2,11], particularly its household or environmental correlates in various geographies [19,20,21,22,23,24] and its contribution to physical activity [25,26,27]. Emerging topics of research include the relationship between children’s travel to school and life satisfaction, mood, or travel satisfaction [28,29,30]. Some research has also been dedicated to evaluating the success of interventions that aim to encourage walking or bicycling to school [31,32]. The focus in transport planning and research on facilitating active travel to school likely stems from a utilitarian view that the school for a child is akin to the workplace for an adult, in terms of a location where children spend much of their time outside of home and that needs to be accessed safely. However, the policy focus on schools has tended to overshadow the needs of children to access a variety of non-school destinations and the quality of the neighborhoods around them [33].

Examining children’s affordances and independent mobility, Kyttä [34] discussed the importance of having diverse destinations that are accessible. However, a quick scan of literature on children’s travel reveals that the focus of most research is on trips to school. Recent growth of the literature on children’s active or independent travel to non-school destinations can help to shift the policy focus for children’s transport planning beyond school-based travel. Researchers have used diverse methodologies to study this topic to date. For example, Bhosale et al. [35] measured children’s independent roaming in New Zealand to 12 locations through a questionnaire to calculate an independent mobility (IM) destination area. Other researchers have created conceptual frameworks [36] or accessibility tools [37] based on travel diaries to categorize children’s travel to various destinations. The use of SoftGIS methods such as mapping activities have also been popular to investigate children’s localized affordances (i.e., a measure of the functional quality of the environment that enables/restricts action) and meaningful places [38,39,40,41]. Children’s activity spaces [42,43] and territorial range [44] also demonstrate how far children travel from home and reveal how children utilize their self-defined neighborhoods. Babb et al. [42] reported that walking trips were most common to home and outdoor spaces, followed by shopping, school, visiting family/friends, and recreation indoors. These studies, and others that are compiled in this scoping review, indicate that children travel to and desire to access places other than home and school. Even children who are not yet school age conceptualize cities as places that should give them access to diverse destinations [45]. Overall, trends indicate that many children are deprived of the opportunity to be independent and to visit a variety of daily destinations.

What is missing from the literature at present is overview of the emerging body of literature examining what types of non-school destinations children access by active or independent travel. A scoping review of existing evidence can identify places that children want or need to access that deserve more attention. This information can help transport planners to design interventions to support children in traveling to places that are important for their well-being. As some countries report variations in active or independent travel among children based on socio-economic and demographic characteristics [46], this information would also be beneficial to clarify factors that influence children’s non-school travel behaviors between geographies.

This scoping review aims to fill these gaps by summarizing quantitative and qualitative research on the topic of active or independent travel to non-school destinations. In addition, it aims to describe relevant methodological approaches that have been employed to better understand children’s travel to non-school destinations. We focus on children and youth under 14 years of age because parents often grant older children greater mobility licenses that permit them to travel farther from home. Based on evidence from studies included in the review, we also categorize non-school destinations by domain of well-being to build a profile of children’s trips. Finally, we identify areas of future research to better center children’s travel needs in transport planning and engage children in creating child-friendly environments.

## 2. Methods

This scoping review was carried out in accordance with the PRISMA-ScR method (Preferred Reporting Items for Systematic reviews and Meta-Analyses extension for Scoping Reviews) [47]. The procedure was reviewed by the research team as well as librarians to ensure that the search terms and strategy were appropriate. The corresponding author can provide the search protocol upon request.

Independent travel to non-school destinations, which includes both quantitative and qualitative approaches, a scoping review is an appropriate type of review for this topic [48]. This is mainly because a systematic review would be limited to quantitative research for this type of review, whereas a scoping review follows a systematic review process while allowing for the inclusion of qualitative research [49]. Therefore, this approach is suitable for emerging research areas where qualitative research is common, such as children’s travel.

### 2.1. Review Question

What is known about elementary aged children’s destinations?

### 2.2. Search Strategy and Eligibility Criteria

Papers were sourced from the following three databases: (i) Web of Science Core Collection; (ii) PubMed; and (iii) APA PsycInfo. We also hand searched three relevant journals related to children and transport: (i) Children’s Geographies; (ii) Journal of Transport & Health; and (iii) Journal of Transport Geography. The literature search included these key terms: (destination* OR “activity space*” OR “important place*” OR “meaningful place*”) AND (independen* OR travel* OR journey* OR roam*) AND child*. We limited our search to peer-reviewed articles published in English between 1980 and July 2021.

Peer-reviewed articles were excluded based on the following criteria:

1. Not peer-reviewed (i.e., book reviews, conference papers or presentations, etc.)
2. Not published in English
3. Published before 1980
4. Population sample was 14 years or older (however, if an article included an age range with some children under 14 years and some over 14 then it was included)
5. Focused solely on children’s active or independent travel to school;
6. Focused solely on motorized trips made with parents or families to school and/or other non-school destinations;
7. A singular methodological focus without describing or identifying empirical findings related to children’s trips;
8. Children’s travel or access to non-school destinations is measured but not reported or described explicitly;
9. The outcome of interest relates to physical activity or other health-related outcomes derived from children’s active or independent travel, and not the child’s travel to a destination or place.

Two authors independently screened the papers for eligibility. We sourced a total of 1293 peer-reviewed articles based on our search strategy and screened all of them by title and abstract to determine eligibility based on the criteria identified above. After this stage, we reviewed 77 articles in full-text and screened further based on content (criteria 4–8). For example, Smith et al. [50] found that active travel to non-school destinations was associated with greater moderate-to-vigorous physical activity, but this study was excluded because the outcome of interest was physical activity and destinations were not discussed in great detail. Likewise, Villanueva et al. [8] explored children’s activity spaces, however this paper was ultimately not included because there was insufficient detail on the local destinations. A total of 27 papers were included in this review from which data were extracted, assessed, and summarized. Following Dijkers’ [51] recommendations, those papers were examined for the two authors and same datasets. Two issues were identified and will be discussed.

#### Data Extraction and Synthesis

We used Covidence, an online software platform for systematic reviews, to organize articles during the title and abstract screening stage. Full-text review and data extraction was conducted in Microsoft Excel. The following items were extracted: Title of the paper, year, first author last name, location, study type, population (age, gender, etc.), inclusion or exclusion criteria, sample of the study, accompaniment status, outcome of interest (what is measured and how it is defined), data collection (instrument used and timeline), methods and analysis, how is a trip or place defined, relevant and main findings (e.g., destinations accessed, CIM, etc.), secondary findings (e.g., gender differences, implications for wellbeing, etc.) and strength and limitations of the study (generalizability, small sample, flaws in method, etc.). We evaluated each study by considering methodological rigor (i.e., eligibility criteria, sample size, sampling strategy, data collection, and risk of bias) and data relevance (e.g., how strongly the reported findings were within scope of the goals of this review). Each study was rated with an overall score of ‘high’ or ‘low’.

## 3. Results

**Figure 1:**
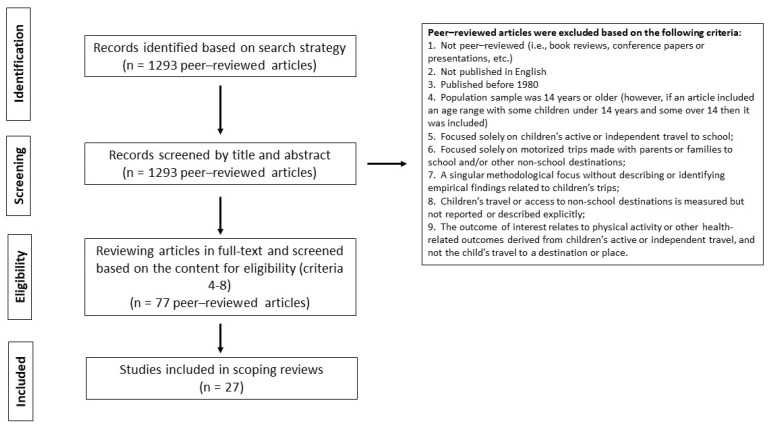
Flowchart of the article selection process.

The results of the search and paper selection are shown in the flow chart (Figure 1). After all steps, 27 papers were retained to respond to the review question.

### 3.1. Overview of Included Papers

The 27 papers considered in this review come from a range of geographic locations (Table 1), though it should be noted that in several cases, more than one paper was published using the same dataset (discussed later). The papers in this review report findings from predominantly urban environments. We categorized the 27 papers according to their outcome of interest: access or travel to places where children spend time (12); activity spaces and territorial range (5); meaningful places or affordances (7); and outdoor play spaces in neighborhoods (3). This section is divided according to those subheadings, where we summarize the empirical findings and describe some of the methodologies of those articles. Table 2 provides a brief overview of these papers.

Several papers used the same dataset: three papers from Bangladesh [52,53,54], two papers from Finland [39,40], two papers from Australia [55,56], one paper from Canada [57] is a subset with additional data from a previous paper [43]. It is important to acknowledge this to avoid distortion of findings that might arise from having multiple publications from the same dataset. In nearly all cases, the research question was distinct as can be seen by the results reported in Table 2.

A few papers from New Zealand used data from a larger study [37,58,59], though each study used a different data source (children’s surveys, parental responses, and children’s interviews).

### 3.2. Access or Travel to Places Where Children Spend Time

The majority of papers we reviewed examine children’s trips to various destinations through travel diaries [37,53,58], surveys/questionnaires [52,55,56,65,66], or mapping activities [36,60,62,68]. However, it should be noted that some of these used the same dataset: 37 and 52, 59 and 66. Some studies used GPS devices to complement data collected from travel diaries [68], while others took a qualitative approach through interviews to explore children’s perceptions of their activities and neighborhoods [60,62]. Several studies collected data through multiple methods, sometimes combining quantitative and qualitative approaches [56,60,62,69].

Studies in this category demonstrate that children across different geographies travel to a diversity of places that support their well-being. Badland et al. [37] analyzed over 3000 trips from 238 students over a 7-day period and found that, apart from school, children commonly access other types of retail, sport facilities, parks, other recreation, and churches. Similarly, Egli et al. [36] had over 1000 children record places that they visited in their neighborhood on a map. Over 2500 destinations were mapped and the most frequently named locations were parks, playgrounds, fields and courts; followed by shops, specifically food shops. The authors used these data to develop the Kids-PoND framework which could be a useful tool for transport planners. In Scotland, Olsen et al. [65] found that 10 to 11-year-old children in their study primarily spent time near their home and school, as well as near a library or place of worship. After adjusting for home and school locations, the authors also reported that children spent more time in small geographic areas containing food and/or drink retail outlets.

With respect to unsupervised trips, the most popular destinations that children accessed independently were a friend or relative’s home, parks or green spaces, recreational facilities, and retail locations [55,56,66,68], although 59 and 66 use the same dataset, so should not be considered distinct results. Access or trips to specific places can differ based on gender [40,52,60]. However, there are some places that children are not able to access. Qiu and Zhu [66] found that few children (less than 5%) were allowed to access other amenities that are important for their physical or mental well-being such as sporting fields or recreation centers, which may reflect parental fears about traffic dangers or distances being too far to travel for children. Similarly, Williams et al. [68] reported that few children (less than 25% in their study) used active modes to travel to places such as retail locations, restaurants, recreational facilities, community centers, or other community locations. Finally, children who completed a travel diary for Chaudhury et al.’s [58] study made few trips to public open spaces which is surprising given that these are often considered ‘child-friendly’ spaces. Again, these findings reinforce the idea of parental fears about traffic danger, distances being too far to travel for children, or inadequate bicycle or pedestrian infrastructure. Overall, there is evidence from multiple countries that children travel alone to many places beyond their school that may presently be overlooked in transport planning decisions for active travel.

Sharmin et al. [52,53] published two papers based on data collected from travel diaries and surveys completed by children in Bangladesh. What is interesting about these studies is that the authors distinguish between discretionary (i.e., exploratory movement without a specific destination in mind) and non-discretionary trips (i.e., wayfinding movement to a specific destination). Most children reported at least one independent trip, with higher rates of independent travel during the week [52]. They also found that children made more independent trips to discretionary destinations, but school was reported as the most frequently visited nondiscretionary destination [53].

Studies that use qualitative methods can shed more light on children’s perceptions of places they frequently access and the value that they attribute to them. Familiarity is an important aspect which affects children’s use of urban spaces and leads them to have positive connotations towards certain local destinations, such as a soccer field close to their school [62]. Children enjoy places based on their function and what they can do in that space [62], which relates to other papers that use affordance theory to understand children’s meaningful places. This is described further in Section 3.3.

### 3.3. Activity Spaces and Territorial Range

In addition to documenting the non-school destinations accessed by children, some studies measure activity spaces or territorial range. The term *activity space* has been defined by Babb et al. [42] as “a measure of the spatial arrangements of travel and the use of urban space to satisfy daily activity needs”. Studies that report on children’s activity spaces generally collect data from GPS devices that are worn over a certain period of time [42,43,61]. By monitoring children’s travel and movement, researchers can establish spatial areas where children spend time. Babb et al. [42] further distinguish between *potential* and *realized* activity spaces where potential indicates the possible activities that may be undertaken but the urban space may be underutilized and realized indicates the capability set for what the child can do.

Related to papers summarized in the previous section, popular destinations for children to access, apart from school, were residential locations of friends and family, places to shop, outdoor spaces, and recreational facilities [42,61]. Two studies reported that children had low levels of independent mobility overall [42,43]. This may be due to small sample sizes, the period of time when data was collected, or household/environmental barriers. Chambers et al. [61] found that children spent more than 50% of their time within 500 m of their home, but that they frequently left their ‘neighborhood’ boundary to go to school, visit other residential locations, and food retail outlets. Babb et al. [42] measured similar activity spaces with children traveling distances up to 3 km, with an average of 432 m and 7 min per trip. Like Brown et al. [60], they also reported gender differences in terms of size of activity spaces and distances traveled by active modes. In a Canadian study by Loebach and Gilliland [43], children spend most of their time out of school either at home or in their neighborhood, which indicates the likelihood of one or more constraints on their mobility (e.g., household or environmental).

In addition to measuring activity spaces, Babb et al. [42] also gave children disposable cameras and instructed them to take photos of aspects of their neighborhood that they liked or hated. This method, known as photovoice, is a useful way to engage children in visualizing and describing their local environment. The authors conducted a thematic analysis of children’s photo-collages by coding elements that were present in the photos such as activities, objects, emotions, and settings. This activity revealed that children with high-realized local activity spaces put more photos of nature and parks in their collages, compared to their peers with low-realized local activity spaces who had more photos of their home and organized sport venues in their collages [42]. Streets were identified as places with positive functioning for children because they created opportunities for socializing and exploring, but they also featured in children’s ‘hate’ collages if they lacked walking paths or had busy roads.

Two studies relate to children’s *territorial range* which is “a measure of how far (in terms of distance or time walking) one could travel from home” [44]. Using the same data from their previous two studies summarized in Section 3.1, Sharmin et al. [54] found that the territorial range for nondiscretionary trips is almost double in length than that of discretionary trips. This makes sense given that travel to a specific destination, such as for coaching activities, would require a child to travel further than if they were roaming freely in their neighborhood. In disadvantaged areas, 37% of children were allowed to roam more than 15 min walk on their own and 50% were allowed to do so with friends [44]. Additional research is needed to compare territorial range among children of different socioeconomic status.

### 3.4. Meaningful Places or Affordances

Mapping activities are a popular method to engage children in identifying places and experiences that are meaningful to them, as well as localized affordances (i.e., a measure of the functional quality of the environment that enables/restricts action). Generally, children are asked to mark any place on an online softGIS survey and indicate why these places are socially, emotionally, or functionally meaningful to them [38]. Children can also indicate whether they traveled to each place independently or using active modes. This allows researchers to compile an extensive, and often large, data set of destinations that children access. For example, children identified over 12,000 locations in one Finnish study [40]. The functional aspect of each place is important to capture because it reveals what children value about places where they spend time and how they use them.

In line with findings from studies measuring territorial range, most of the children’s meaningful places in Finnish studies are located within 500 m from home [40,67]. Compared to Finnish children, Japanese children mark around 75% of their meaningful places within 1 km of home [41]. This highlights that there are cultural differences in children’s access to non-school destinations or differences in built environments. In one of the earliest studies using softGIS mapping activities, children marked more places that allowed them to ride a bicycle, play ball games, or run [40]. This indicates, at least in the Finnish context, that children highly value places that support their physical and social well-being. Places associated with leisure activities such as computer use, shopping, and playing sports were also commonly identified by children [40]. The authors also found land use differences between categories of children’s meaningful places: children located more emotional experiences and action activities in green spaces, whereas social activities and leisure-time places were in more dense areas. This somewhat mirrors findings from another Finnish study where children reported more active travel trips in green spaces and residential areas and fewer trips in dense or traffic-dominated urban environments [67]. There is also evidence that children’s mobility licenses and the independence that they are granted by parents may influence the places that they are allowed to access unsupervised [38].

Walk-along interviews with children have also been conducted to understand how children perceive their neighborhoods and articulate what they like or dislike about the places they spend time. Chaudhury et al. [59] held 140 child-led interviews starting from home to explore children’s experiences of public open spaces. They found that over half of the children were allowed to go to public open spaces in their neighborhood unsupervised, but that distance, stranger danger, and traffic constrained their independent mobility. Children perceived neighborhood parks to be engaging with a high level of affordances, meaning opportunities for experiences, particularly their interactions with trees in public open spaces [59].

### 3.5. Outdoor Play Spaces in Neighborhoods

The final category of papers that were reviewed pertain to outdoor play spaces for children, which can either be public or private. Two studies utilized maps and interviews to understand children’s use of neighborhood places for independent outdoor play [57,63,64]. Neighborhood parks are traditionally viewed as the main place where children play, but these studies revealed other places that children use for exploration and play. Furneaux et al. [64] learned that children in Toronto, Canada prefer to play in the back alleys behind their home rather than their backyards because the communal space offers more opportunities for social interactions and games with other children. Children enjoy neighborhood parks because of the activities that they can do, and greatly enjoy being outdoors [57,63]. However, Ergler et al. [63] highlight a few challenges that arise in environments that are not designed to be child-friendly. First, the authors report that the “invisibility of city children playing on the wide sidewalks… normalize(s) year-round indoor play”. Second, urban playgrounds that do not offer sufficient interesting or valuable activities can be a barrier to promoting independent outdoor play for children. More research is needed to explore where children play outdoors and how they conceptualize a ‘play space’.

## 4. Discussion

This scoping review summarized findings from 27 studies to provide a comprehensive overview of the non-school destinations or places that children access by active or independent travel. Overall, there is evidence from multiple countries that children can be independently mobile to a variety of places that are important for their well-being (see Table 3). These studies highlight various popular destinations where children spend time that should be prioritized more by transport planners including retail locations, libraries, community centers, and various types of green spaces. Children living in urban environments generally make most of their trips within 1 km of their home [42,61] and identify most of their meaningful places within that territorial range as well [40,41,67]. Our paper demonstrates the importance of facilitating children’s travel to places beyond school and neighborhood parks, as well as the importance of designing child-friendly environments that offer a diversity of amenities and places within 1 km of residential areas.

The built environment where children spend the majority of their time outside of school, which likely encompasses both the home and surrounding neighborhood, can facilitate or constrain opportunities for play, social interaction with peers, exploration, travel, and physical activity [55]. In the absence of adult supervision, children can learn from various interactions with their surroundings and experiences with other people when they travel or play independently. However, our scoping review highlights that dominant cultures of mobility and planning are constraining children’s mobility which can negatively impact their well-being. A few studies that we reviewed explicitly reported that children were limited in trips to certain places [58,66,68] or uninterested in places designed for children such as local parks [63]. We found that children in some countries can primarily access places that relate to physical or social well-being (e.g., local parks or green spaces, recreational facilities) more than places that support other domains of well-being (e.g., libraries, community centers, retail locations). These results may be due to how some countries perceive certain places as being more appropriate or relevant for children to access than others. It has also been noted that parental perceptions of safety [55] or heavy traffic in neighborhoods [70], social concerns [16], potential stranger danger [58], traffic along the route [52,58], and inequities in access to amenities based on income [52] may limit the children’s travel. These are a few primary root causes that limit children’s mobility which need to be addressed to support their travel or access to destinations that support their well-being.

Children’s constrained mobility to certain places or with outdoor play are issues present in urban areas worldwide which points to a broader pattern in planning that fails to consider children’s affordance needs when designing for active travel or independent outdoor play. Based on the findings from our scoping review, we argue for researchers and planners to focus less on *how* children travel and more on *which* places children need to access to support their well-being and development. They should identify *where* children spend their time or play beyond their home or school to understand how they benefit from these destinations and to examine whether barriers exist to limit their travel. Table 3 is a profile of children’s trips to non-school destinations which can be a valuable resource for future researchers and transport/urban planners. Child-friendly environments are typically conceptualized at the neighborhood or city level [68], therefore these groups at the local level should investigate the state of children’s current active or independent travel to these places to determine areas for improvement. It would also be worthwhile to quantify the benefits related to well-being that children gain from their access to non-school destinations to measure changes at the neighborhood or city level over time.

Affordance theory has been employed increasingly in the literature due to Kyttä’s research (see [39,67]), but it should also have a place in urban and transport planning practice. Various design principles that children need in urban environments have already been advanced in the existing literature such as proximity and child scaling [71]. Another recent review summarized socio-physical factors of child-friendliness including access, safety, children’s participation, and independent mobility [72]. We found some evidence of these indicators in certain countries where children have access to green space and local environments, as well as a certain degree of independent mobility, proximity/walkability, and the ability to actualize affordances. However, the *diversity* and *quality* of destinations that children can access, as well as the impacts of travel on children’s well-being, are criteria that appear to be continually overlooked in planning. Jansson et al. [72] note that access in the context of child-friendly environments often relates to implementing solutions that address traffic which often enables or hinders mobility for children. The application of affordance theory to urban and transport planning and practice can help these fields shift from focusing primarily on route-based factors, (e.g., separating and reducing traffic) towards a more comprehensive approach to facilitating children’s travel that includes the provision of functional opportunities to make the environment supportive for their well-being. In particular, the conceptual framework of children’s neighborhood destinations developed by Egli et al. [36] is useful because it incorporates activities and qualities that children associate with destinations that they visit. As affordance theory has been primarily employed in Scandinavian studies, we recommend that researchers in other regions of the world apply it in combination with softGIS surveys, particularly those with limited research on children’s travel to non-school destinations.

Studies that engage children in innovative ways go one step further than those that employ surveys or questionnaires because children are not restricted by a predetermined list of non-school destinations when asked where they travel to. This enables researchers to learn specifically where children spend their time and why, which can inform local transport planning decisions. With respect to methodological approaches, we wish to highlight how mapping activities [36,37] and softGIS surveys [38,39,40,41,67] can generate sizable data sets of rich information regarding children’s mobility to places that are socially, emotionally, or functionally meaningful for them. Walk-along interviews [59] and photovoice [42] additionally offer children the opportunity to be heard by adults and to vocalize or illustrate how they conceptualize a ‘place’ or ‘neighborhood’. This is one of the benefits of conducting a scoping review—this qualitative evidence would have been excluded if we had pursued a systematic review instead. Other novel mapping tools, such as Paper2GIS [73], and photo elicitation approaches such as photo-journeys [74] could also be adapted for research with children.

There are some limitations to our work. Research exists where non-school destinations are recorded in the paper, but as it was not the focus of the research, such a keyword may not have been in the title or abstract. We also acknowledge that additional literature on this topic is likely published in languages other than English. For this reason, there may be relevant papers that were missed in our literature search and that were not included in the review based on the eligibility criteria for inclusion.

### Future Research

We encourage future research on this topic to clearly report and discuss the non-school destinations that children access. As previously mentioned, we found that the literature seems to be more focused on *how* children traveled to places as opposed to *where* they were going. Many papers that we screened in full-text were ultimately excluded from this review because they were not clear about the types of non-school trips made by children (for example, [69]) or the non-school destinations were a secondary focus of the paper (for example, [8,75]).

Research examining children’s perceived and actualized freedom of movement and exploration to non-school destinations is more limited which is currently a gap in the literature. Future studies should also investigate how children, planners, and parents/guardians rate their neighborhoods or cities according to various socio-physical indicators of child-friendly environments as identified by Jansson et al. [72], considering the quality of access and proximity to non-school destinations that they experience. Potential discrepancies should be addressed and evaluated over time to provide an assessment of the overall child-friendliness of specific urban areas. Finally, there is a need for a synthesis of findings from studies published in languages other than English, which is a limitation of our work. More studies in English language, especially international comparisons, and studies focused on children in rural areas would additionally extend our knowledge on this topic.

## 5. Conclusions

In addition to home and school, children travel to various places including a friend or relative’s home, local parks or green spaces, recreational facilities, and different retail locations (e.g., restaurants). Children in some countries can access a greater diversity of destinations than others, which is likely due to environmental or cultural factors that influence active travel or independent mobility. Most studies engage children in data collection, and sometimes their parents/guardians too, which is encouraging given the importance of children’s participation in research. Researchers should continue to employ new or innovative methods for engaging children in identifying where they travel or how they perceive their local environment. In addition to the directions for future research described above, we also recommend greater involvement from parents and community organizations to identify the root causes that limit children’s mobility based on local contexts and to promote children’s involvement in neighborhood or city planning consultations.

## Figures and Tables

**Table 1:** Articles reviewed and included by region and country.

Region	Countries	Number of Articles
Asia	Japan Bangladesh	1 3
Europe	Denmark Finland Scotland United Kingdom	1 4 1 1
North America	Canada United States	4 2
Oceania	Australia New Zealand	4 6

**Table 2:** Summary of peer-review articles included in the scoping review.

Country	Authors and Year	Category	Methods	Sample (N) and Age	Main Results	Data Evaluation
Australia	Babb et al., 2017 [42]	Activity spaces	Survey GPS devices Photovoice	N = 49 9–13 years	Most children had constraints on their mobility; 27% of reported trips were made by active modes of transport; Children walked to home, school, shops, recreation areas, and to visit friends or family	High
New Zealand	Badland et al., 2015 [37]	Access or travel to places where children spend time	Travel diary	N = 238 9–11 years	Common destinations accessed by children were primary schools, other types of retail, sport facilities, parks, other recreation, and churches	High
Finland	Broberg et al., 2013 [38]	Meaningful places and affordances	softGIS survey	N = 901 9–14 years	Younger children were accompanied to a meaningful place for more trips and travelled more by active travel compared to older children who travelled further from home	High
Finland	Broberg et al., 2013 [39]	Meaningful places and affordances	softGIS survey	N = 1837 10–15 years	Affordances reached alone (i.e., unsupervised) included places to be alone, on computers, and with animals; children were accompanied by an adult to see show, go to a museum, or spend time with other adults	High
United Kingdom	Brown et al., 2008 [60]	Access or travel to places where children spend time	Questionnaire Interviews Map Annotations	N = 1009 9–12 years	Boys were more likely to travel to all places alone than girls were, especially for trips to the park and shops; Girls were more likely to be accompanied to places such as the cinema, shops, parks, shopping centers, and sports facilities	High
Australia	Carver et al., 2014 [44]	Territorial range	Questionnaire Accelerometers	N = 271 8–15 years	37% of children were allowed to roam more than 15 min walk on their own and 50% were allowed to roam with friends	High
New Zealand	Chambers et al., 2017 [61]	Activity spaces	Cameras GPS devices	N = 114 11–13 years	Children spent more than 50% of their time within 500 m of their home but left their ‘neighborhood’ boundary to go to school and visit other residential locations or food retail outlets	High
New Zealand	Chaudhury et al., 2017 [58]	Access or travel to places where children spend time	Travel diary	N = 240 9–12 years	Only 2.10% of recorded trips were made to public open spaces (POS) and 1.08% were trips made to POS independently	Low
New Zealand	Chaudhury et al., 2019 [59]	Meaningful places or affordances	Go-along walking interviews	N = 140 9–13 years	Over half of suburban children were allowed to travel independently to a public open space compared to children from inner-city neighborhoods	Low
Denmark	Christiansen et al., 2015 [62]	Access or travel to places where children spend time	Group mapping workshop Interview	N = 17 11–12 years	The main reasons for children liking places were related to the function of the place; Familiarity affects how children perceive and use urban spaces	High
Australia	Christian et al., 2015 [55]	Access or travel to places where children spend time	Survey	N = 181 8–15 years	40% of children travelled independently to a friend’s or another family member’s house; 48% to a park, oval, or sporting field; 30% to the local shop; and 29% to at least 3 of these local destinations	High
New Zealand	Egli et al., 2020 [36]	Access or travel to places where children spend time	Online interactive mapping survey	N = 1102 7–13 years	2559 neighborhood destinations were mapped by children; the most frequently places were: parks, playgrounds, fields and courts; and food shops	High
New Zealand	Ergler et al., 2013 [63]	Outdoor play spaces in neighborhoods	Mapping activity	N = 20	Children enjoy many activities in neighborhood parks and being outdoors	Low
Canada	Furneaux & Manaugh, 2019 [64]	Outdoor play spaces in neighborhoods	Interviews Mapping activity	N = 12 9–13 years	Children enjoy playing in large parks near their home, their schoolyard, and in the back alleys behind their homes	High
Finland	Kyttä et al., 2012 [40]	Meaningful places or affordances	softGIS survey	N = 1837 10–15 years	Children located 12,343 meaningful places; common functional meanings identified by children at the action level were bicycling, playing ball games, and running	High
Finland Japan	Kyttä et al., 2018 [41]	Meaningful places or affordances	softGIS survey	N = 1341	In Japan, 75% of meaningful places were within 1 km from home, while in Finland this was significantly less (53%); educational, commercial, natural, and traffic land uses were more popular among girls, while recreational, religious, and other places were more popular among boys	High
Canada	Loebach & Gilliland, 2014 [43]	Activity spaces	SurveyGPS devices	N = 143 9–13 years	Most children (86.7%) had low levels of independent mobility and spend over 75% of their time in their neighborhood activity space when not in school	High
Canada	Loebach & Gilliland, 2016 [57]	Meaningful places or affordances	GPS devices Survey Interviews	N = 23 9–13 years	Children who had higher levels of independent mobility and larger activity spaces also had higher perceived levels of local affordances to neighborhood destinations	High
United Kingdom	Olsen et al., 2019 [65]	Access or travel to places where children spend time	Survey	N = 100 10–11 years	Children spend their time primarily near their home or school, but also spend time in places near a library or place of worship	High
United States	Qiu & Zhu, 2021 [66]	Access or travel to places where children spend time	Survey Google Street View Audits	N = 525 8–11 years	Common destinations accessed by children were a friend or relative’s home in the neighborhood, neighborhood streets, parks, and playgrounds	High
Finland	Sarjala et al., 2015 [67]	Meaningful places and affordances	softGIS survey	N = 1037 10–14 years	Most trips were made independently either with friends (65%) or alone (28%); Over half of all identified meaningful places were located closer than 1 km from home	High
Bangladesh	Sharmin et al., 2020 [52]	Access or travel to places where children spend time	Questionnaire	N = 151 10–14 years	Children made more independent trips on weekdays (70.6%) compared to weekends (29.4%); common destinations included school and parks	Low
Bangladesh	Sharmin et al., 2021 [53]	Access or travel to places where children spend time	Travel diary	N = 151 10–14 years	Children were more independent to discretionary destinations (1.15 trips/child) than nondiscretionary destinations (1.02 trips/child)	Low
Bangladesh	Sharmin et al., 2021 [54]	Territorial range	Questionnaire	N = 151 10–14 years	Children’s territorial range for nondiscretionary trips is almost double the territorial range for discretionary trips	Low
Australia	Villanueva et al., 2013 [56]	Access or travel to places where children spend time	Survey Mapping activity	N = 1132 10–12 years	Both boys and girls reported more trips to green spaces, friends’ houses, and shops	High
Canada	Williams et al., 2018 [68]	Access or travel to places where children spend time	GPS devices	N = 388 10–13 years	Common destinations accessed by active travel were home, school, other people’s homes, and parks or greenspace	High
United States	Yoon & Lee, 2019 [18]	Outdoor play spaces in neighborhoods	Survey	N = 3449	Cultural differences between Hispanic and White children exist in play spaces with Hispanic children reporting fewer places to play	High

**Table 3:** Popular non-school destinations accessed by children categorized by domain of well-being based on the work of Waygood et al. [10].

Domain of Well-Being	Destination
Physical (i.e., physical activity, exercise)	Park Recreational facility Sporting field Public open space
Psychological (i.e., mental or emotional health)	Library Community center Place of worship
Cognitive (i.e., learning, exploring)	Library Park Public open space Community center
Social (i.e., social interactions, social capital, social skills, connections to community)	Friend’s house Relative’s house Library Park Public open space Community center Place of worship Retail location Restaurants
Economic	Retail location

## Data Availability

All materials and data associated with this integrative review are available in an online repository.
